# Subjective Duration Distortions Mirror Neural Repetition Suppression

**DOI:** 10.1371/journal.pone.0049362

**Published:** 2012-12-12

**Authors:** Vani Pariyadath, David M. Eagleman

**Affiliations:** 1 Department of Neuroscience, Baylor College of Medicine, Houston, Texas, United States of America; 2 Department of Psychiatry, Baylor College of Medicine, Houston, Texas, United States of America; The University of Plymouth, United Kingdom

## Abstract

**Background:**

Subjective duration is strongly influenced by repetition and novelty, such that an oddball stimulus in a stream of repeated stimuli appears to last longer in duration in comparison. We hypothesize that this duration illusion, called the temporal oddball effect, is a result of the difference in expectation between the oddball and the repeated stimuli. Specifically, we conjecture that the repeated stimuli contract in duration as a result of increased predictability; these duration contractions, we suggest, result from decreased neural response amplitude with repetition, known as repetition suppression.

**Methodology/Principal Findings:**

Participants viewed trials consisting of lines presented at a particular orientation (standard stimuli) followed by a line presented at a different orientation (oddball stimulus). We found that the size of the oddball effect correlates with the number of repetitions of the standard stimulus as well as the amount of deviance from the oddball stimulus; both of these results are consistent with a repetition suppression hypothesis. Further, we find that the temporal oddball effect is sensitive to experimental context – that is, the size of the oddball effect for a particular experimental trial is influenced by the range of duration distortions seen in preceding trials.

**Conclusions/Significance:**

Our data suggest that the repetition-related duration contractions causing the oddball effect are a result of neural repetition suppression. More generally, subjective duration may reflect the prediction error associated with a stimulus and, consequently, the efficiency of encoding that stimulus. Additionally, we emphasize that experimental context effects need to be taken into consideration when designing duration-related tasks.

## Introduction

The perceived duration of a stimulus is modulated by repetition and novelty. Consequently, the first stimulus in a stream of repetitions appears expanded in comparison to successive ones [1]. Similarly, any oddball stimulus in such a visual train appears dilated in duration, an illusion called the oddball effect [2] [3] [4] [5] [6]. We and others have suggested that subjective duration is tied to the amplitude of the neural response to a stimulus [7] [8] [9] [10]. In such a framework, with repetition the amplitude of the neural response diminishes – a phenomenon called repetition suppression [11] [12] [13] [14]. Therefore, the perceived duration of a repeated stimulus should contract and thus, in comparison, the first presentation of the stimulus or any novel stimulus would appear dilated in duration.

In this paper, we test the above hypothesis by focusing on some characteristics of repetition suppression and probing for their perceptual correlates in the oddball effect. First, repetition suppression has been shown to increase with increasing number of presentations, until it hits saturation by around 6–8 presentations [12]. If the oddball effect truly rides on repetition suppression, one would expect that the perceived duration of a stimulus should contract more and more with the first 4–5 repetitions. When the oddball arrives in the visual train, the subject may compare the oddball either to the stimulus just preceding it or to the average duration of all preceding stimuli; in either scenario, the size of the oddball effect should depend on the number of repetitions preceding it. Specifically, the oddball effect should increase in size with increasing number of repetitions preceding the oddball.

## Materials and Methods

### Participants

All participants were between 18–45 years of age and had normal or corrected-to-normal vision. Research was approved and all participants were consented in writing according to the procedures of the Institutional Review Board at Baylor College of Medicine.

### Stimuli

Stimuli consisted of a blue line (subtending a visual angle of 11.04×0.87°) that was presented at the center of the screen against a black background. On each trial, a blue line (of a fixed orientation) repeatedly appeared for 500 ms with an interstimulus interval of 300 ms. After a certain number of repetitions, an oddball line – a line that was rotated at an certain angle from the standard line – would appear followed by more standard lines. The oddball line was presented for different physical durations ranging from 300 to 700 ms (in steps of 50 ms). On each trial, 7 lines, including the oddball, appeared one after the other. At the end of the trial, participants were required to respond to whether the oddball stimulus was longer or shorter in duration than the standard lines. The next trial began 1000 ms following their response.

## Results

### Experiment 1

**Figure 1:**
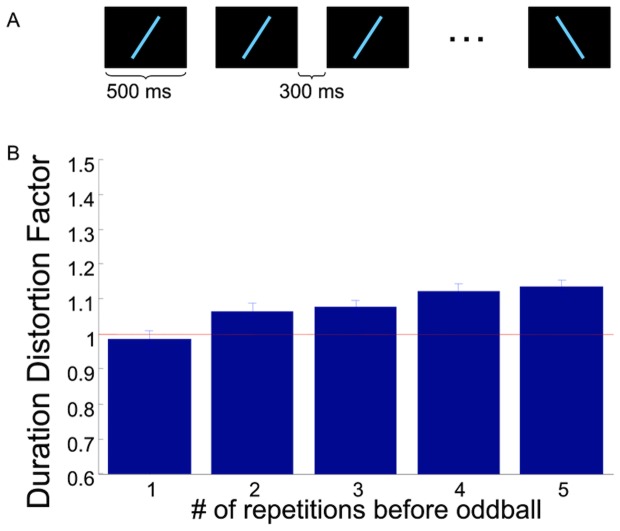
The size of the oddball effect depends on the number of repetitions of standard stimulus. (A) Cartoon depicting the experimental design. Participants viewed a stream of repeated lines with an oddball line that appeared anywhere from the 2^nd^ to the 6^th^ position. (B) The number of repetitions of the standard stimulus modulates the size of the temporal oddball effect.

In this experiment, the oddball line, which was always rotated 45° away from the standard, could appear anywhere from the 2nd through the 6th position in a serially-presented visual train (Figure 1A). The orientation of the standard line was randomized. Data was collected in 2 blocks of 140 trials each with the 5 conditions (the position of the oddball) randomly interleaved.

11 participants ran the experiment. Psychometric curves were fit to each participant's data to quantify the point of subjective equality (PSE) of the standard and oddball stimuli, i.e. the point at which the participant reported the oddball was longer than the standard 50% of the time. From this PSE, the duration distortion factor (DDF) was calculated as the ratio of the standard duration to the PSE for the oddball [3] The average DDF for the oddball line at each of the 5 possible positions is presented in Figure 1B.

As is evident from Figure 1, the size of the oddball effect depends on the number of repetitions of the standard stimulus. A one-way repeated measures ANOVA showed a significant effect of number of repetitions (F_4,40_ = 18.496; p<2.86e-06, corrected for sphericity using the Huynh-Feldt correction). Further, we tested a linear mixed model in R using the lmer function [15] with a random intercept and a fixed slope, with an F-test with Satterwaite's approximation of degrees of freedom. This established that the size of the oddball effect increases significantly with the number of repetitions (F_1,18_ = 56.9; p<2e-16). This effect of number of repetitions is reminiscent of repetition suppression [12] and supports our hypothesis.

### Experiment 2

Neural activity suppression can occur not only as a result of repetition, but also when a non-repeated stimulus is predictable [16]. In other words, the amplitude of the neural response can reflect the expectedness of a stimulus and the consequent improved efficiency in encoding it. Given this, we hypothesized that the more prediction-error there is for an oddball stimulus, or the farther in coding-space it is from the standard, the greater its perceived duration relative to the standard stimuli.

**Figure 2:**
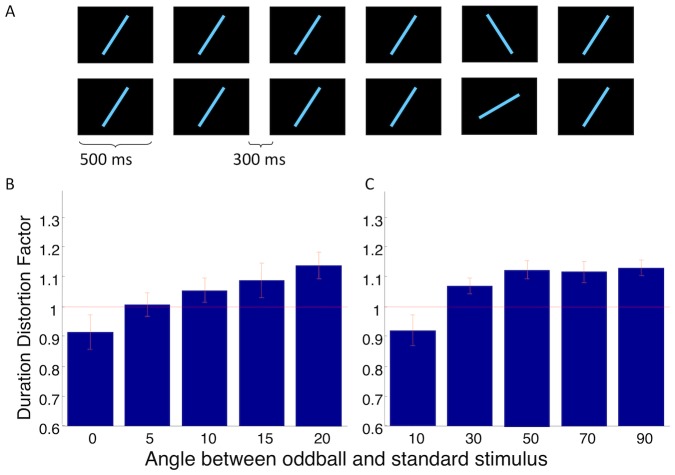
The size of the oddball effect depends on the degree of novelty between the oddball and the repeated stimulus. (A) Cartoon depicting two possible trials. Top: the oddball is very different compared to the standard; bottom: oddball is very similar to the standard. (B) The size of the oddball effect increases linearly with the difference in angle between the oddball and standard stimuli. (C) The effect of novelty saturates by ∼50° of difference.

To address whether the degree to which the deviance of the oddball from the standard stimulus modulates the oddball effect, we varied the difference in angle between the oddball and the standard lines (Figure 2A). This experiment was conducted in 2 ways: in one, the difference in angle between the oddball and the standard lines was varied by 0, 5, 10, 15 or 20 degrees, and in the second, this difference was varied by 10, 30, 50, 70 or 90 degrees. The orientation of the standard line on each trial was randomized. The oddball appeared at the 5th position in the stimulus sequence. Nine and 16 subjects participated in part A and B of the experiment respectively. As in the previous experiment, participants reported whether the oddball line was longer or shorter in duration than the standard lines.

We found that the size of the oddball effect varied significantly with the degree of novelty of the oddball line relative to the standard line (Figure 2B,C; F_4,28_ = 21.77; p<7.78e-07 corrected for sphericity, and F_4,60_ = 10.05; p<0.00078 corrected for sphericity, one-way repeated measures ANOVA). The size of the oddball effect increased monotonically with the degree of novelty from 0 to 20 degrees (F_1,22.5_ = 7.79; p<0.01). This finding is consistent with those of Schindel et al (2011), who also demonstrated that oddball effects scaled with the angular discrepancy between repeated and oddball stimuli. Further, one-sampled t-tests against mean 0 on the slope of the increase from 10°–50° was statistically significant (t_15_ = 3.34; p<0.01), but not from 50°–90° (t_15_ = 0.32; p = 0.75), indicating that the effect of novelty appears to saturate by about 50° of difference (Figure 2C).

### Experiment 3

**Figure 3:**
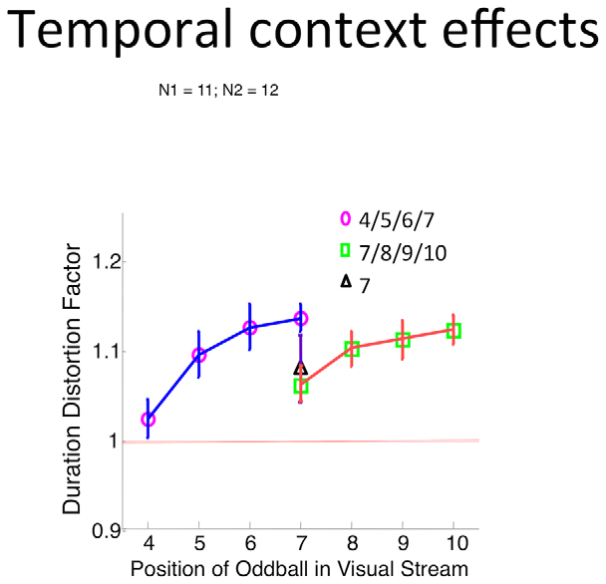
The oddball effect is subject to experimental context. (A) The oddball effect at the 7^th^ position was tested in 3 different blocks – among interleaved trials in which the oddball appears between the 4^th^ and 7^th^ position, interleaved trials in which the oddball appears between the 7^th^ and 10^th^ position, and trials in which the oddball only appeared at the 7^th^ position. (B) The size of the oddball effect when the oddball appears at the 7^th^ position is different depending on the expectations within the experimental block of trials.

From Figure 2B and C, it is evident there is a difference between the oddball effect sizes for 10° oddballs in blocks A and B (t_23_ = 2.1045; p<0.05) even though the stimuli are identical in both cases. This difference is suggestive of an experimental context effect on duration distortion. In light of this, we next asked whether the temporal context of the oddball (its position in the sequence) might also influence the duration distortion. To address this question, we varied the position of the oddball in the visual train as shown in Figure 3, but in 3 distinct blocks: in one, the oddball could appear at the 4th, 5th, 6th, or 7th positions; in a second block, the oddball appeared at the 7th, 8th, 9th or 10th positions; and in the third block, the oddball appeared only at the 7^th^ position. In all cases, the oddball was the final stimulus in the visual train. We predicted that the size of the oddball effect at the 7th position would differ between the blocks as a result of a different temporal context.

Results are shown in Figure 3. As in Figure 2, we found an experimental context effect. Specifically, the size of the oddball effect differs at the 7th position depending on the temporal context in which this condition was presented. The DDF at the 7th position is larger in the 4/5/6/7 block than in 7/8/9/10 block (Figure 3, t_21_ = 2.97, p<0.008). There was no statistical difference between the DDF in the block in which the oddball always appeared at the 7^th^ position relative to the other two blocks (t_23_ = 0.9593, p = 0.3474 and t_24_ = −0.7182, p = 0.4795).

## Discussion

Our data indicate that both the number of repetitions of the standard stimuli preceding the oddball and the relative novelty of the oddball play a considerable role in determining the size of the oddball duration effect. The novelty findings (Figure 2) are consistent with Schindel et al [6], who have independently demonstrated that the distinction between the oddball and the standard stimulus is critical to the size of the oddball effect. From these results, a consistent model emerges: with each repetition of the stimulus, a more energy-efficient representation is acquired. Concurrently, the perceived duration of the stimulus contracts, and thus an oddball or unexpected stimulus appears longer in comparison [3] [8] [9] [10]. In this framework, oddballs that are more similar to the standard stimuli still benefit from the predictive encoding, and thus do not trigger as large a response as vastly dissimilar oddballs. If our model is correct, subjective duration may potentially inform us how efficiently a stimulus has been encoded.

While we have explored the immediate effects of repetition here, repetition also involves long-term, familiarity-like effects [11]. Our experimental design used essentially the same stimulus presented in different orientations for standard and oddball stimuli, and is therefore not well suited to address the question of long-term repetition effects. (See [17] for an exploration of this aspect of repetition suppression; the author showed that the long-term effects of repetition do not seem to influence duration in the same manner as immediate repetition.)

Other researchers have suggested attentional explanations for the oddball effect [2]. In that framework, the appearance of the oddball necessitates an increase in attentional resources and thereby seems expanded in duration. However, we have previously shown that increasing the emotional salience of the oddball does not result in larger oddball effects; instead, it is simply the violation of prediction that appears to be important [3]. It is parsimonious to assume that predictive coding lies at the heart of the duration oddball effect, and that exogenous attention is drawn as a *consequence* of its violation [6] [9].

It is worth noting that participants were instructed to judge the duration of the oddball with respect to the stimuli that come before it; they may, therefore, be doing one of two things: 1) comparing the duration of the oddball to the standard stimulus immediately preceding it, or 2) comparing the duration of the oddball to the average duration of all the standard stimuli preceding it in the trial. These comparison strategies may vary from individual to individual. Regardless of how the participant is carrying out the comparison process, our interpretation of the data remains the same. The size of the oddball effect for an individual may vary based on the strategy employed, but the effect of number of repetitions or of the degree of novelty should be present in qualitatively the same fashion for all participants.

We also found that the experimental context—that is, the set of possible oddball positions within the experiment—strongly influences the oddball effect. A similar effect was seen in Tse et al [2]: the authors used randomly interleaved trials of different standard durations and found that the oddball appeared to be contracted in duration for stimulus durations under 150 ms. This contraction effect is not surprising when taking the experimental context into account: the size of the oddball effect on any given trial depends on the range of oddball sizes seen during the course of the experiment. We suspected that the duration contraction effect at brief duration would disappear or that a normal oddball effect would reappear if the short duration condition were tested in isolation. In our hands, when measuring the oddball effect for a standard duration of 70 ms (with oddballs varying from 30 to 110 ms), no duration distortions were found (data not shown).

Why is subjective duration affected by experimental context? For Experiment 2, one framework for understanding this is found in Helson's adaptation level theory, in which stimulus judgments are influenced by the range of previously encountered stimuli [18]. Although duration judgments for the oddball were always made relative to the same, standard stimuli in our experiments, participants presumably built up probability distributions from the oddball durations in the previous trials. As for Experiment 3, an alternative suggestion is also possible: the temporal context effect seen in Figure 3 is reminiscent of a hazard rate function, i.e., the probability of an event occurring given that it has not occurred thus far [19]. This probability can have strong implications for the amplitude of the neural response in sensory areas [20]. For example, when visual stimuli are presented at uncertain delays, climbing activity has been observed in visual association areas whose amplitude corresponds with the length of waiting period leading up to the onset of the visual stimulus [20] [21]. The amplitude of such climbing activity would be maximal in the case of the oddball at the 7^th^ position in the 4/5/6/7 condition, and minimal in the 7/8/9/10 condition. Our neural response amplitude hypothesis [9] predicts that climbing activity representing hazard-like computations are added on to oddball stimulus representations (which are then compared to that for the repeated stimuli) and distorts the size of the oddball effect.

In summary, our results provide a prediction to be tested using electrophysiology: the degree of duration distortion of an oddball stimulus mirrors neural response magnitudes, which is itself modified by predictive coding and experimental context.
